# Association Analysis between Genetic Variants of *elovl5a* and *elovl5b* and Poly-Unsaturated Fatty Acids in Common Carp (*Cyprinus carpio*)

**DOI:** 10.3390/biology11030466

**Published:** 2022-03-18

**Authors:** Yan Zhang, Qing-Song Li, Yu-Qing Ye, Qi Wang, Xiao-Qing Sun, Ran Zhao, Jiong-Tang Li

**Affiliations:** 1 National Demonstration Center for Experimental Fisheries Science Education, Shanghai Ocean University, Shanghai 201306, China; zhangy@cafs.ac.cn (Y.Z.); m190110454@st.shou.edu.cn (Q.-S.L.); yeyuqing95@163.com (Y.-Q.Y.); 2 Key Laboratory of Aquatic Genomics, Ministry of Agriculture and Rural Affairs, Chinese Academy of Fishery Sciences, Beijing 100141, China; wangqi@cafs.ac.cn (Q.W.); sunxiaoqing@cafs.ac.cn (X.-Q.S.); zhaoran@cafs.ac.cn (R.Z.); 3 Beijing Key Laboratory of Fishery Biotechnology, Beijing 100141, China

**Keywords:** common carp, poly-unsaturated fatty acid, fatty acid elongase, association study, genomic selection

## Abstract

**Simple Summary:**

PUFAs have an essential impact on human health, but their availability constitutes a critical bottleneck in food production. Although fish is the traditional source of PUFAs, it is limited by the stagnation of fisheries. Many studies aim to increase the PUFA products of fish. Genetic markers are efficient in aquaculture breeding. Fatty acid desaturase 2 (*fads2*) and elongase 5 (*elovl5*) are the rate-limiting enzymes in the synthesis of PUFAs. The allo-tetraploid common carp is able to biosynthesize endogenous PUFAs. However, selective breeding common carp with high PUFA contents was hindered due to a lack of effective molecular markers. For future breeding common carp capable of producing endogenous PUFAs more effectively, we previously identified the polymorphisms in the coding regions of two duplicated *fads2*, *fads2a* and *fads2b*. However, the polymorphisms in the duplicated *elovl5*, *elovl5a* and *elovl5b*, were not detected. This study screened the genetic variants in the coding regions of *elovl5a* and *elovl5b*. Moreover, the joint effects of multiple coding SNPs in *fads2b* and *elovl5b*, two major genes regulating the PUFA biosynthesis, were evidenced with the increased explained percentages of the PUFA contents. These polymorphisms in these two genes were used to evaluate the breeding values of PUFAs. These SNPs would be potential markers for future selection to improve the PUFA contents in common carp.

**Abstract:**

The allo-tetraploid common carp, one widely cultured food fish, is able to produce poly-unsaturated fatty acids (PUFAs). The genetic markers on the PUFA contents for breeding was limited. The polymorphisms in *elovl5a* and *elovl5b*, the rate-limiting enzymes in the PUFA biosynthesis, have not been investigated yet. Herein, we identified one coding SNP (cSNP) in *elovl5a* associated with the content of one PUFA and two cSNPs in *elovl5b* with the contents of eight PUFAs. The heterozygous genotypes in these three loci were associated with higher contents than the homozygotes. Together with previously identified two associated cSNPs in *fads2b*, we found the joint effect of these four cSNPs in *fads2b* and *elovl5b* on the PUFA contents with the increased explained percentages of PUFA contents. The genotype combinations of more heterozygotes were associated with higher PUFA contents than the other combinations. Using ten genomic selection programs with all cSNPs in *fads2b* and *elovl5b*, we obtained the high and positive correlations between the phenotypes and the estimated breeding values of eight PUFAs. These results suggested that *elovl5b* might be the major gene corresponding to common carp PUFA contents compared with *elovl5a*. The cSNP combinations in *fads2b* and *elovl5b* and the optimal genomic selection program will be used in the future selection breeding to improve the PUFA contents of common carp.

## 1. Introduction

Poly-unsaturated fatty acids (PUFAs) with at least 18 carbons [1] play physiologically important roles and are essential for human health because they are the major components of complex lipid molecules involved in numerous critical biological processes [2,3,4]. In general, fish are the main available source of long-chain PUFAs (LC-PUFAs, fatty acids with at least 20 carbons [5]) for human dietary [6]. In diploid fish, fatty acid desaturase 2 (*fads2*) and elongase 5 (*elovl5*) are two rate-limiting enzymes in the PUFA biosynthesis pathway [7,8]. Therefore, the polymorphisms in these two genes are hypothesized to be associated with the PUFA contents. Indeed, the polymorphisms of *fads2* in bovine and fish were reported to be significantly associated with the PUFA content [9,10,11]. Genome-wide association studies revealed that single nucleotide polymorphisms (SNPs) of *elovl5* were associated with omega-6 (n-6) and omega-3 (n-3) fatty acid (FA) levels in human, sheep, and bovine [12,13,14]. Identifying the mutants in *fads2* and *elovl5* in fish of economic value would benefit for the future breeding fish having high contents of PUFAs.

The allo-tetraploid common carp is widely cultured in the world and is able to convert dietary 18-Carbon PUFAs to LC-PUFAs including arachidonic acid, eicosapentaenoic acid, and docosahexaenoic acid [7,15]. As an allo-tetraploid fish, it maintains the tetraploidization status and encodes almost twice that the diploid Cyprininae fish. Previously we cloned two *fads2* genes (*fads2a* and *fads2b*) and identified the polymorphisms in these two genes associated with the PUFA contents in common carp [9]. These findings not only supported that there existed two homoeologues of *fads2* but also suggested that the PUFA biosynthesis pathway in common carp was more complex than diploid Cyprininae fish. Although two *elovl5* genes (*elovl5a* and *elovl5b*) in common carp were sequenced [16], the questions of whether there exist the polymorphisms in these two genes and whether these polymorphisms are associated with the PUFA contents have not been studied.

Recent study revealed that two subgenomes in common carp performed balancing of differential expression in response to different conditions, dampening the stimulus impact to the expression of the duplicated genes [17]. As for the PUFA biosynthesis, which of two duplicated *elovl5* genes is the major effect gene is still unknown. Furthermore, how duplicated *fads2* genes and duplicated *elovl5* genes coordinate to regulate the PUFA biosynthesis is less studied.

To answer these questions, in this work, we sequenced the coding regions of common carp *elovl5a* and *elovl5b* and detected the polymorphisms in these two genes. With the association study, we identified the SNPs significantly associated with the PUFA contents and found more associated SNPs in *elovl5b* than *elovl5a*. We further examined the joint effects of the associated SNPs in *fads2b* and *elovl5b* on the PUFA contents. Finally, we obtained the high and positive correlations between the contents and the predicted breeding values of eight PUFAs using the cSNPs in these two genes. These cSNPs would be used as the biomarkers to facilitate the selective breeding of common carp with high PUFA contents. 

## 2. Materials and Methods

### 2.1. Sampling and Measuring PUFA Contents

We collected the juveniles of three bred strains of common carp, including ‘HuangHe’ (HHC) strain, ‘FuRui’ (FRC) strain and ‘Jian’ (JC) strain in May 2018, described in our previous study [9]. These strains were sampled from different provinces of China and had different morphology traits, which were shown in detail before. We had cultivated these juveniles for one year in one pond at the Chinese Academy of Fishery Sciences (Fangshan, Beijing, China) with the same commercial diet. In May 2019, we randomly selected 124 individuals of FRC, 98 JC fish, and 47 HHC fish. The tissue collection, liver RNA extraction, reverse-transcription of RNA to cDNA, and the muscles PUFA content calculation were described before. 

Briefly, FAs were converted into the fatty acid methyl esters (FAMEs), which were further extracted and purified by thin-layer chromatography following the strategy of Li et al. [18]. A total of 25 types of FAs were identified using the 7890A GC System (Agilent Technologies, Wilmington, Delaware, USA) by comparing their GC retention time with the time of the peaks of a Supelco 37 Component FAMEs standard mix (Nu-chek Prep Inc., Elysian, MN, USA). The relative proportion of each among 4 types of 18-Carbon PUFAs and 8 types of LC-PUFAs was calculated as (area of one PUFA/total area of 25 types of FAs) × 100. 

### 2.2. Sequencing and Genotyping

Based on the reference full-length sequence of *elovl5a* and *elovl5b* in common carp (MK893918.1 and MK893919.2), the gene-specific primers were designed to amplify the complete coding sequence (CDS) regions (Supplementary Table S1). PCR amplification was carried out according to the protocol as described previously [9]. The annealing temperatures were set as 56 °C and 60 °C, respectively. After sequencing the PCR products with the Sanger method, we aligned them to the reference sequences of *elovl5a* and *elovl5b* using Blastn, respectively [9]. Theoretically, one sequence expected to be from *elovl5a* should have a higher identity value to *elovl5a* than *elovl5b* and vice versa. To call cSNPs, the confirmed sequences were aligned to the corresponding reference sequences using the novoSNP software [19]. The SNPs were identified with F-scores ≥ 30, and the homozygotes and heterozygotes were auto-detected with this software. If a site in one individual had one sequencing peak, then this site was homozygous. If this site had two peaks, it was heterozygous. If this site in one sample had three or more peaks, it was discarded. 

### 2.3. Genetic Diversity of Common Carp elovl5a and elovl5b

The genetic distances and population structures among three strains were calculated with all retained genotypes in *elovl5a* and *elovl5b* together. The genetic distances and the population structures of all samples were calculated with Tassel 5 [20] and the admixture function [21] of the package LEA [22] in R 4.1.0, respectively. The first two eigenvectors of the PCA result were plotted. The population structures (K value ranging from 2 to 6) were displayed with pophelper v2.3.1 [23].

We grouped three strains into one population and calculated the genetic diversities of the cSNPs, which had a frequency over 0.04. The diversity indicators included the observed heterozygosity (Ho), the expected heterozygosity (He), the minor allele frequency (MAF), and the polymorphism information content (PIC). The former three indicators were measured with the Genepop software 4.7 [24]. The PIC was estimated using PICcalc 0.6 [25]. Using TBtools [26], we classified the effects of cSNPs on the coding sequences into the stop loss, stop gain, non-synonymous substitution, and synonymous substitution. We also calculated the linkage disequilibrium (LD) between any two SNPs in each gene. The LD was measured using the LDheatmap function in R [27] and represented with D’ value. We clustered cSNPs into one haplotype block if the D’ of any two compared cSNPs in this gene was over 0.8. 

### 2.4. Associations of cSNPs in elovl5a and elovl5b with the Contents of 12 PUFAs

To identify the cSNPs in these two genes associated with the PUFA contents, the general linear model (GLM) and the analysis of variance (ANOVA) were used to study the association between each PUFA content and the genotypes, respectively. We ran the GLM model with the parameters of the genetic distance matrix and 100,000 permutations using Tassel 5 [20]. This method was widely applied in the association study between the polymorphisms in candidate genes and the phenotypes [28,29]. To perform ANOVA, we classified all individuals into different groups based on their genotypes in one SNP. The pairwise comparison between any two groups on each PUFA content with ANOVA. We corrected the ANOVA *p* values using the false discovery rate (FDR) method for multiple hypothesis testing. One cSNP was deemed to be significantly associated with one PUFA content when it had a *p* value < 0.05 in the GLM method and an FDR-corrected *p* value < 0.05 in the ANOVA. The explained percentage of phenotypic variation (PV) of each cSNP was measured using Tassel 5.

### 2.5. Joint Effects of Significant SNPs in elovl5b and fads2b on the PUFA Contents

Previously, we identified one cSNP in *fads2a* associated with the content of C20:3n-6 PUFA. Another two cSNPs in *fads2b* were significantly associated with the contents of seven PUFAs and six PUFAs, respectively. Herein, we identified three cSNPs associated with the contents of multiple PUFAs. Since *fads2b* and *elovl5b* were two major effect genes on the contents of multiple PUFAs, we hypothesized that the joint analysis of multiple associated SNPs could detect a larger effect than single SNP and identify the optimal genotype combinations associated with higher PUFA contents. Hence, we estimated the joint effects of four significantly associated SNPs (two in *fads2b* and two in *elovl5b*) on the contents of multiple PUFAs. We generated different genotype combinations from all these SNPs. If one genotype combination was observed in at least three individuals, this combination was used in the comparison. For each PUFA, we performed the pairwise comparisons of the PUFA contents among different retained combinations using ANOVA in R software. The explained percentages of PV of the genotype combination to the content of each PUFA was estimated with the function of ‘lm’ [30] in R.

### 2.6. Estimating the Breeding Values with the cSNPs in elovl5b and fads2b on the PUFA Contents

Further, we were interested in whether all identified cSNPs in *fads2b* and *elovl5b* would be applied into estimating the breeding values (BVs) of the PUFA contents. The cSNPs with MAFs over 0.03 were used to estimate the BVs with BWGS [31]. This package integrates multiple programs available for the genomic BV prediction, including GBLUP [32], EGBLUP [33], Ridge regression (RR) [34], LASSO [35], Elastic Net (EN) [36], Bayesian ridge regression (BRR) [37], Bayesian LASSO (BL) [38], Bayes A (BA) [39], Bayes B (BB) [40], and Bayes C (BC) [41]. We used each program to estimate the BV of each PUFA in each validated individual. The maximum proportion of missing value for filtering marker column was set as 0.2 with the minimum allele frequency for filtering markers as 0.03. We performed 20 independent replicates in the cross validation for each program on each PUFA. First, in each replicate of one cross validation, these individuals were separated into the reference group and the validation group, respectively. The randomly sampled 90% of all individuals having the amplified sequences from both *fads2b* and *elovl5b* were treated as the reference group to train the breeding models and the remaining 10% of all individuals as the validation group. For each PUFA, the BV of each individual in the validation group by each program was represented as the predicted content. Further, in each replicate of one cross validation by each program, we calculated the Pearson correlation coefficient value (CV) between the actual contents and the predicted contents of each PUFA across individuals in the validation group. Second, we calculated the mean BV of each PUFA and its standard deviation (SD) of all individuals predicted by each program during 20 replicates. Third, to estimate the BV accuracy of each program, we calculated the mean CV and its SD of each program for each PUFA during 20 replicates. The mean squared error of prediction (MSEP) and corresponding standard deviation (SD-MSEP) were also computed.

## 3. Results

### 3.1. Genetic Diversities of Common Carp elovl5a and elovl5b

We confirmed that the entire CDS regions of *elovl5a* and *elovl5b* were successfully sequenced in 204 and 269 individuals, respectively. Both amplified lengths of *elovl5a* and *elovl5b* cDNA sequences were 876 bp, corresponding to seven exons. The base contents of A, G, C, and T in the *elovl5a* CDS were 28.9%, 24.5%, 21.4%, and 25.2%, respectively. For *elovl5b*, the base contents of A, G, C, and T in the CDS were 25.9%, 25.4%, 23.0%, and 25.4%, respectively. These two reference mRNAs were highly identical with a similarity of 95%, higher than that of common carp *fads2a* and *fads2b* (89.86%) [9]. 

Ten cSNPs including one non-synonymous cSNP (ns-cSNP) and nine synonymous cSNPs (s-cSNP) were identified in six exons of *elovl5a* (Table 1 and Supplementary Table S2). These cSNPs had 25 genotypes where five SNPs had three genotypes per locus. The MAFs of the cSNPs ranged from 0.0147 to 0.3769 with seven SNPs having MAF less than 0.1. The Ho values of ten cSNPs were from 0.0294 to 0.4724 where the Ho values of five cSNPs were smaller than 0.1. The He values of ten cSNPs were from 0.029 to 0.4697 and six cSNPs had He values smaller than 0.1. The PIC values of these SNPs had a range from 0.0286 to 0.3594. All the MAF, Ho and He values of five cSNPs, half of SNPs in *elovl5a*, were lower than 0.1. These data suggested their low polymorphic levels.

Eight cSNPs including three ns-cSNPs and five synonymous ones were identified in five exons of *elovl5b* (Table 1 and Supplementary Table S3). We found 20 genotypes in these eight cSNPs, four of which had three genotypes per locus. The Ho, He, PIC, and MAF values of these SNPs were in the ranges of 0.1413~0.9071, 0.1313~0.4988, 0.1227~0.3744, and 0.0706~0.4758, respectively. Comparing the diversities of these two genes revealed that *elovl5b* had higher polymorphic levels than *elovl5a*. Although three cSNPs in *elovl5b* were observed with MAF smaller than 0.1, all SNPs had PIC, Ho, and He higher than 0.1, supporting the higher polymorphisms in *elovl5b*. 

These three common carp strains were grouped together based on all genotypes in *elovl5a* and *elovl5b* in the PCA analysis (Supplementary Figure S1A). The first two eigenvectors accounted for 41.61% and 10.76% of the total genetic variances. The population structures, plotted with different K values using all genotypes in *elovl5a* and *elovl5b*, also suggested that three strains had similar genetic components (Supplementary Figure S1B). These two data revealed that there were no strain-specific SNPs in *elovl5a*/*elovl5b*. Hence, these three strains were grouped into one population in the association study.

### 3.2. cSNPs in elovl5a and elovl5b Associated with the PUFA Contents

**Figure 1:**
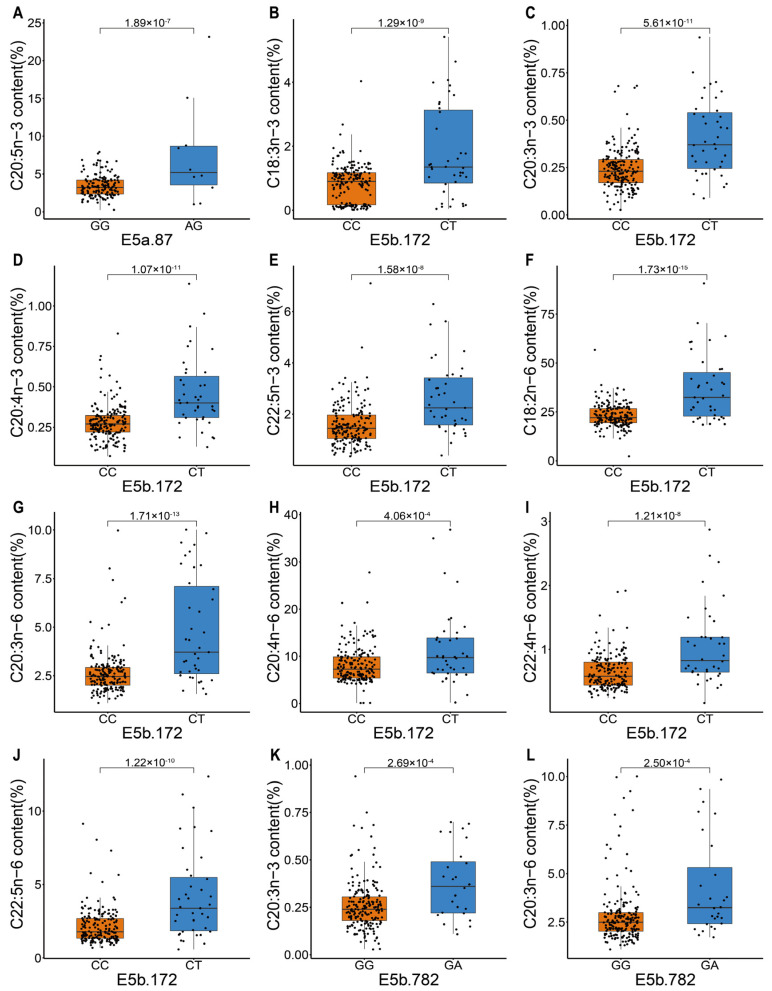

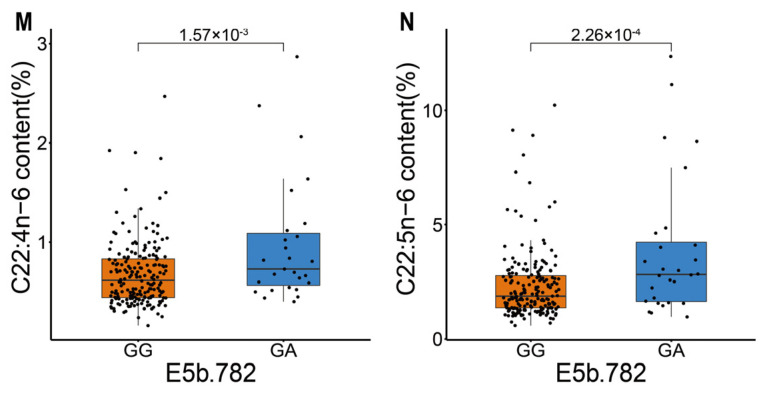
Box plots showing significant PUFA content differences between two genotypes at three loci, E5a.87, E5b.172, and E5b.782. The corrected *p* values were calculated with ANOVA. (**A**): PUFA content values of genotype GG and AG atE5a.87 locus; (**B**–**J**): PUFA content values of genotype CC and CT at E5b.172 locus; (**K**–**N**): PUFA content values of genotype GG and GA at E5b.782 locus.

Three cSNPs, one in *elovl5a* (E5a.87) and two in *elovl5b* (E5b.172 and E5b.782), were identified to be associated with the contents of multiple PUFAs. In these three loci, we only observed the homozygous wild-types and the heterozygous genotypes while the homozygous mutations were not found. The synonymous SNP in *elovl5a* was significantly associated with the C20:5n-3 PUFA content (Table 2 and Figure 1A). The heterozygote of this SNP corresponded to higher content of C20:5n-3 than the homozygote with a fold change of 2.19.

Two ns-cSNPs in *elovl5b*, E5b.172 and E5b.782, were significantly associated with several PUFA contents by using both GLM and ANOVA (Table 2). The ns-cSNP of E5b.172 led to the mutation from proline to serine. This SNP was significantly associated with nine PUFAs contents, including four n-3 PUFAs (C18:3n-3, C20:3n-3, C20:4n-3, and C22:5n-3) and five n-6 PUFAs (C18:2n-6, C20:3n-6, C20:4n-6, C22:4n-6, and C22:5n-6) (Figure 1B–J). The heterozygote had higher contents of the above nine PUFAs than the homozygote. The fold changes of the mean contents of the associated PUFAs were from 1.43 to 2.13 (Table 2). The ns-cSNP of E5b.782 led to the mutation from arginine to glutamine. It was associated with only four PUFAs contents, including C20:3n-3, C20:3n-6, C22:4n-6, and C22:5n-6 (Figure 1K–N). Likewise, the heterozygote of this SNP had higher contents of the above four PUFAs than the homozygote. The fold changes of the mean associated PUFA contents were from 1.43 to 1.66 (Table 2). The contents of four PUFAs (C20:3n-3, C20:3n-6, C22:4n-6, and C22:5n-6) were associated with both cSNPs. 

Two haplotype blocks were detected in each of the CDS regions of *elovl5a* and *elovl5b*, respectively (Supplementary Figure S2). E5b.172 and E5b.782 were distributed in two different blocks, respectively. The contents of four PUFAs (C20:3n-3, C20:3n-6, C22:4n-6, and C22:5n-6) were associated with both cSNPs in *elovl5b*. These data suggested these two cSNPs might coordinate to regulate the biosynthesis of these four PUFAs. However, the SNP of E5b.172 was associated with the contents of the other five PUFAs, suggesting that this cSNP solely affected these PUFAs contents. Since the cSNP of E5b.172 had more associated PUFAs and explained more percentages of PV than E5b.782 (except C20:3n-6), suggesting that the former might be the major SNP. 

### 3.3. Joint Effect of elovl5b and fads2b on the PUFA Contents

The cSNPs in *elovl5b* were associated with more PUFA contents than that in *elovl5a*, suggesting that *elovl5b* might be the major effect gene regulating the PUFA biosynthesis in common carp. Previously, we found that *fads2b* might be another major gene responding to common carp PUFA contents and that two cSNPs in *fads2b* were associated with the contents of multiple PUFAs [9]. 

**Figure 2:**
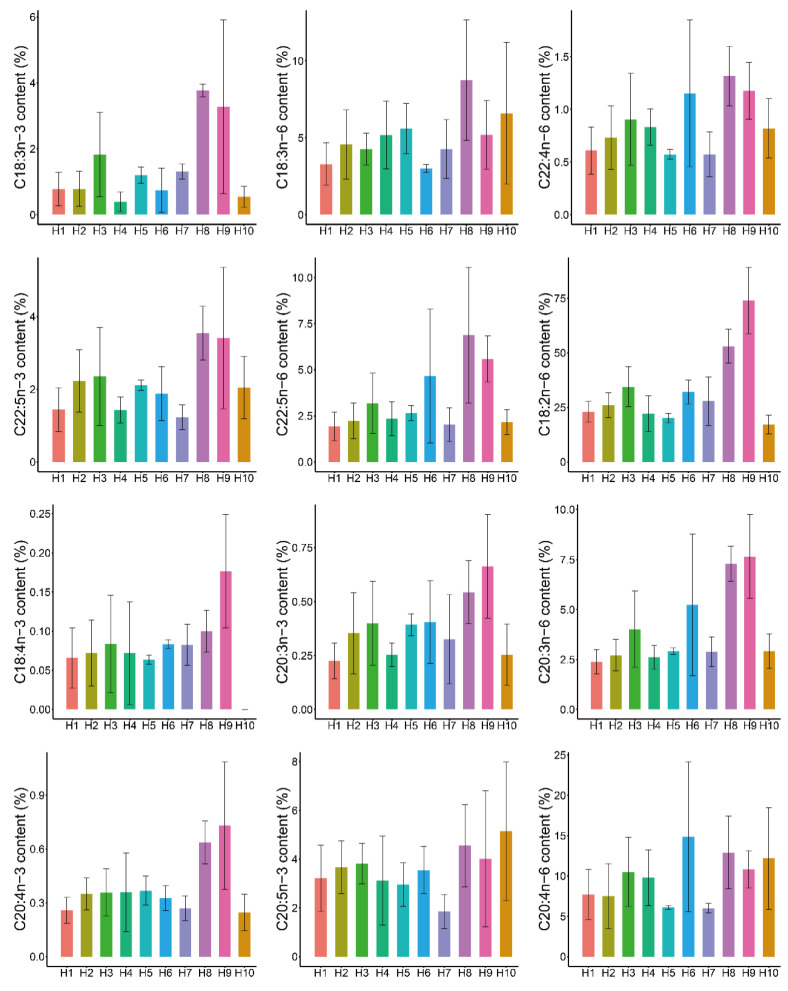
Bar plots showing the mean contents of PUFAs among ten genotype combinations. The genotype combination information and corresponding PUFA contents were shown in Supplementary Table S4. The bar heights represent the mean contents of PUFAs except the outliers. The whiskers are the standard deviations.

Interestingly, the cSNPs in *fads2b* were associated with four n-3 PUFAs (C18:3n-3, C20:3n-3, C20:4n-3, and C22:5n-3) and four n-6 PUFAs (C18:2n-6, C20:3n-6, C22:4n-6, and C22:5n-6). The cSNPs in *elovl5b* had significant association with four n-3 PUFAs (C18:3n-3, C20:3n-3, C20:4n-3, and C22:5n-3) and five n-6 PUFAs (C18:2n-6, C20:3n-6, C20:4n-6, C22:4n-6, and C22:5n-6). The cSNPs in these two genes were involved in the contents of eight common PUFAs, suggesting the coordinative regulation of the PUFA biosynthesis through the mutations in *fads2b* and *elovl5b*. Hence, we estimated the joint effects of four significantly associated cSNPs in two major effect genes (two in *fads2b*, fads2b.751 and fads2b.1197; and two in *elovl5b*, E5b.172 and E5b.782) on the contents of 12 PUFAs. Theoretically, we should observe 16 genotype combinations with four cSNPs. In practice, among 223 individuals having the genotypes of both *fad2b* and *elovl5b*, ten types were detected in at least three individuals (Figure 2 and Supplementary Table S4). The most frequent combination (H1), where all four loci were the homozygotes of the reference bases, was observed in 162 individuals. The other combinations were observed in much fewer individuals. We did not detect the combination including four homozygotes of the mutation bases.

The multi-cSNPs combinations greatly improved the explained percentages of PV compared with the single SNP (Table 3). For three PUFAs (C20:3n-3, C20:3n-6, and C22:5n-6), the contents had four associated SNPs in both two genes. For C20:3n-3, the explained percentages of PV by each SNP ranged between 2.15~13% while the percentage increased to 32.73% with the combination of four cSNPs. For C20:3n-6 and C22:5n-6, the genotype combination improved the explained percentages to 37.59% and 28.19%, respectively. For three PUFAs (C20:4n-3, C22:5n-3, and C22:4n-6), the contents had three associated SNPs in both these two genes. The genotype combinations also increased the explained percentages to 33.22%, 22.63%, and 21.26%, respectively. Although there were only two associated cSNPs in *fads2b* and *elovl5b* in C18:3n-3 and C18:2n-6, the SNP combination explained 33.6% and 54.95% of PV, respectively, much higher than single SNP. For the C20:4n-6 PUFA content, only E5b.172 was associated with 2.1% of PV whereas the combination of four cSNPs had 13.06% of PV. Intriguingly, for the remaining three PUFAs (C18:4n-3, C18:3n-6, and C20:5n-3) having no associated SNPs in these two genes, the genotype combination also contributed to the percentages of PV with a range between 4.24%~13.06%. The increased explained percentages of PV indicated the joint effects of these four cSNPs on the contents of 12 PUFAs.

The joint effect analysis also clearly showed which genotype combination had higher contents of PUFAs than the other combinations. For eight PUFAs having the associated cSNPs in both *fads2b* and *elovl5b*, the genotype combination with higher heterozygous levels corresponded to higher contents of PUFAs. The combination (H8) including four heterozygotes corresponded to the highest contents of five PUFAs including C18:3n-3, C18:3n-6, C22:4n-6, C22:5n-3, and C22:5n-6 (Figure 2 and Supplementary Table S4). The genotype combination corresponded to higher contents of five PUFAs than the homozygote combination (H1) with fold changes ranging between 2.16 and 4.83. Another combination (H9) including three heterozygotes and one homozygote corresponded to the highest contents of five PUFAs including C18:2n-6, C18:4n-3, C20:3n-3, C20:3n-6, and C20:4n-3 (Figure 2 and Supplementary Table S4). This combination corresponded to higher contents of these five PUFAs than the homozygote combination (H1) with fold changes between 2.57 and 3.22. Taken together, these two genotype combinations could be effective markers to select common carp of high PUFAs contents.

### 3.4. The cSNPs in fads2b and elovl5b to Predict the Breeding Values

Because the cSNPs in *fads2b* and *elovl5b* had joint effects on the PUFA contents, we tried to answer whether these cSNPs had the potentials to be used for selection breeding. A total of 35 cSNPs were detected in *fads2b* and *elovl5b*. The MAFs of 25 cSNPs were over 0.03 and hence were used to estimate the BVs. These 25 cSNPs existed in 223 individuals. Ten programs in BWGS tool provided the prediction results (Supplementary Table S5). 

**Figure 3:**
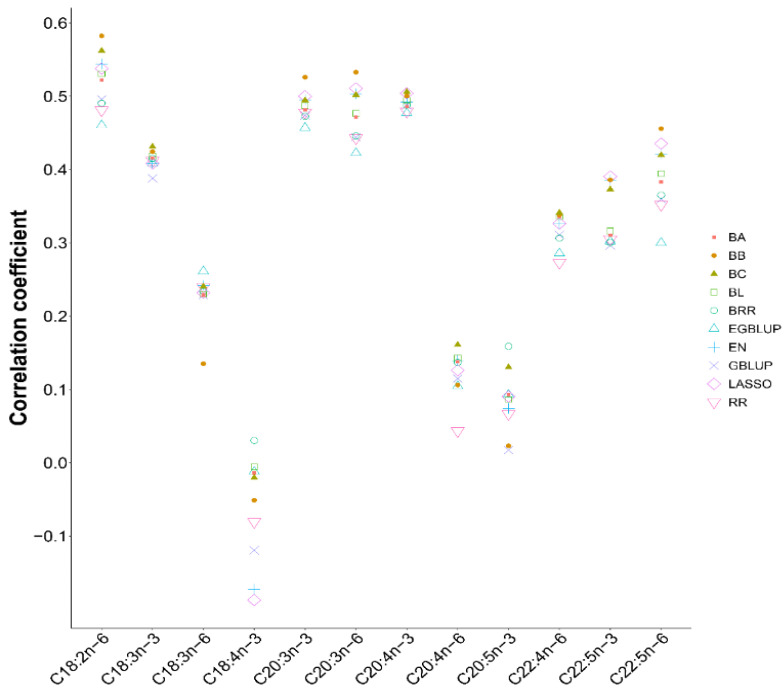
Distribution of the mean Pearson correlation CVs for each PUFA using ten methods with 25 cSNPs in *fads2b* and *elovl5b*. The CV was calculated between the actual contents and the predicted contents of each PUFA across individuals in the validation group.

Since there was only one cSNP or no cSNP in these two genes associated with the contents of C18:3n-6, C18:4n-3, C20:5n-3, and C20:4n-6, the highest mean CV between the actual contents and the BVs were only 0.26. Even in C18:4n-3, the mean CVs of nine programs except BRR were negative (Figure 3). However, for the remaining eight PUFAs having at least two cSNPs in these two genes, the BB program had the best mean CVs in C18:2n-6 (0.5824), C20:3n-3 (0.52605), C20:3n-6 (0.53285), and C22:5n-6 (0.4558). For C18:3n-3 and C 22:4n-6, the BB program was the second-best tool with slight lower CVs (0.4245 and 0.33805) than the top best tool, BC (0.43135 and 0.3413). Likewise, for 22:5n-3, the performance of the BB program also ranked the second with a slight lower CV (0.38595) than the best tool, LASSO (0.39055). For C20:4n-3, the CV of BB program (0.50045) was much close to that of the top best (LASSO, 0.50395) and the second-best tool (BC, 0.50605). These results indicated that the cSNPs in *fads2b* and *elovl5b* could be used to predict the selection breeding potential of high contents of eight PUFAs and that the BB program would be the optimal tool for breeding if taken all eight PUFAs into consideration.

## 4. Discussion

The availability of PUFAs has become important in food production. Although fish is the traditional source of PUFAs, fish PUFA product is limited. Many studies aim to increase fish PUFA contents. Although feeding different ingredients could improve the fish PUFA content [42], this strategy is feed-consuming. This goal could be alternatively achieved by breeding fish capable of biosynthesizing endogenous PUFAs more effectively. Genetic markers were evidenced to be useful in selective breeding of aquaculture species. To date, few effective molecular markers associated with high PUFA contents were identified, hindering the selective breeding of common carp with high PUFA contents. Therefore, it would be necessary to develop the genetic markers applicable to breeding.

The species-specific whole genome duplication and parallel subgenome evolution in common carp generated two homoeologues of *fads2* (*fads2a* and *fads2b*) and two homoeologues of *elovl5* (*elovl5a* and *elovl5b*). We explored that *fads2b* was the major effect gene associated with the common carp PUFA contents compared with *fads2a* [9]. However, the question which gene in two duplicated *elovl5* genes makes more contributions to the PUFA biosynthesis has not been answered yet. *Elovl5b* was associated with more PUFAs and had higher explained percentage of PVs than *elovl5a*, suggesting that the former might be the major effect gene to improve the PUFA contents. More functional studies including yeast heterologous expression system and expression patterns of these two genes are required for further validation of their effects.

In our current study and previous study [9], three strains of common carp showed no significant inter-strain genetic differences in *fads2a*/*fads2b*/*elovl5a*/*elovl5b*, demonstrated by both PCA and population structure analysis. The individuals from one strain were grouped together with the samples from the other strains, suggesting that they might be sampled from multiple families. The grouping also increased the genetic diversities of the studied population and improved the statistical power of the association analysis

We observed one synonymous cSNP in *elovl5a* associated with one PUFA content. One possible reason is that this synonymous cSNP might lead to the changes in DNA methylation and gene expression of *elovl5a*. In humans, the polymorphisms in the FADS region modify the epigenetic methylation, which further regulate the fatty acid metabolism [43] and contribute to the non-alcoholic fatty liver disease [44]. Whether this cSNP possibly modifies the *elovl5a* DNA methylation needs further research. Two ns-cSNPs in *elovl5b* were associated with several PUFA contents. The enzymes activity would be affected by either amino acid mutations in the active sites [45] or distal mutations site away from the active site [46]. Thus, we speculate that E5b.172 and E5b.782 lead to amino acid change and further possibly changed the elongase activities. 

In our previous study, the heterozygote advantage on the PUFA contents was observed in the polymorphisms of *fads2b* [9]. Herein, we confirmed the heterozygote advantages in the polymorphisms in *elovl5a* and *elovl5b*. We did not find the homozygotes of the minor alleles in both *elovl5a* and *elovl5b*. The heterozygotes of three identified cSNPs corresponded to higher PUFA contents than the homozygotes of the reference bases, suggesting that the heterozygosity of *elovl5a* and *elovl5b* might increase their elongase activities. We also revealed the heterozygote advantage in the genotype combination from *fads2b* and *elovl5b*. The combinations of H8 and H9 having four or three heterozygotes had higher contents of the PUFAs than the homozygote combination.

How duplicated *fads2* and *elovl5* genes coordinate to regulate the PUFA biosynthesis is less studied. Our studies showed that *fads2b* and *elovl5b* were two major effect genes on the PUFA contents compared with *fads2a* and *elovl5a*, respectively. The cSNPs in *fads2b* and *elovl5b* were associated with the contents of eight common PUFAs. However, in six PUFAs, fads2b.751 made higher explained percentages of PVs than the cSNPs in *elovl5b* (Table 3), suggesting that improving the fatty acid desaturase activity would be more efficient to increase the PUFA contents than the elongase activity. 

None has studied the joint effects of multiple SNPs on the PUFA contents. The explained percentages of PVs by four cSNPs were higher than the sum percentage of each SNP, indicating the additive effects by these cSNPs on the PUFA contents. The coordination to improve the PUFA contents required the simultaneous mutations in these two genes. We also used the cSNPs in these two genes to predict the BVs of the PUFA contents. The best prediction accuracies of the contents of eight PUFAs ranged between 0.3413 and 0.5824. These values were in the BV reliability range from 0.04 to 0.72 for economic traits in sheep, pig, and cattle [47,48,49].

We comprehensively investigated the polymorphisms in *fads2a*, *fads2b*, *elovl5a*, and *elovl5b*. Although *fads2* and *elovl5* were two rate-limiting enzymes in the PUFA biosynthesis [18], few cSNPs in *fads2a* and *elovl5a* were identified and four cSNPs in *fads2b* and *elovl5b* explained 21.26~54.95% of PVs for the contents of eight PUFAs, suggesting that there might exist other regulatory elements or genes involved in the common carp PUFA biosynthesis. The SNPs in the promoter region of *elovl5* significantly related to fatty acid content in many animals [13,50,51,52,53,54]. Moreover, it was reported that *elovl4*, *elovl6*, and other genes also participated in the PUFA biosynthesis [51,54,55]. Thus, it is necessary to scan the polymorphisms in the promoter of *elovl5a* and *elovl5b* and the genomic regions of the related genes and then identify their associations with the PUFA contents in the future.

## 5. Conclusions

We identified the polymorphisms in the CDSs of two duplicated common carp *elovl5*, *elovl5a* and *elovl5b*. The association study identified three cSNPs in these two genes significantly related to the PUFA contents in common carp. *Elovl5b* might be the major gene regulating common carp n-3 and n-6 PUFA biosynthesis, and two ns-cSNPs in this gene might be the main effect SNPs. The joint effects of four cSNPs in *fads2b* and *elovl5b* improved the explained percentages of PVs of the PUFA contents. The individuals having more heterozygotes of four cSNPs had higher PUFA contents than the ones having the homozygotes, suggesting that the former would be used as the parents for selective breeding of offspring having higher PUFA contents. The cSNPs in these two genes could be applied to estimating the breeding values of the PUFA contents with the optimal tool of the BB program. In sum, our results highlight the importance of the polymorphisms in *elovl5a* and *elovl5b*, other critical factors in the PUFA biosynthesis in common carp. These cSNPs would be useful markers for selection to improve the PUFA contents in common carp.

## Figures and Tables

**Table 1:** Genetic diversities of SNPs in the coding sequences of *elovl5a* and *elovl5b*.

Locus	Gene	Position */Exon	Ho	He	PIC	MAF	Genotype	Amino Acid Change
E5a.29	*elovl5a*	29/1	0.1225	0.1649	0.1513	0.0907	CC	T-I
							CT	
							TT	
E5a.87	*elovl5a*	87/2	0.0493	0.0480	0.0469	0.0246	AG	L-L
							GG	
E5a.180	*elovl5a*	180/2	0.1029	0.0976	0.0929	0.0515	AC	S-S
							CC	
E5a.351	*elovl5a*	351/4	0.0735	0.0708	0.0683	0.0368	CC	Y-Y
							CT	
E5a.429	*elovl5a*	429/4	0.0588	0.0843	0.0808	0.0441	CC	H-H
							CT	
							TT	
E5a.552	*elovl5a*	552/5	0.4608	0.4538	0.3508	0.348	CC	Y-Y
							CT	
							TT	
E5a.582	*elovl5a*	582/5	0.1569	0.2001	0.1801	0.1127	AA	P-P
							AG	
							GG	
E5a.651	*elovl5a*	651/6	0.0294	0.029	0.0286	0.0147	AA	T-T
							GA	
E5a.798	*elovl5a*	798/7	0.0837	0.0802	0.077	0.0419	AG	S-S
							GG	
E5a.810	*elovl5a*	810/7	0.4724	0.4697	0.3594	0.3769	AA	I-I
							AT	
							TT	
E5b.172	*elovl5b*	172/2	0.1822	0.1656	0.1519	0.0911	CC	P-S
							CT	
E5b.174	*elovl5b*	174/2	0.1413	0.1313	0.1227	0.0706	CA	P-S
							CC	
E5b.195	*elovl5b*	195/2	0.2156	0.1924	0.1739	0.1078	AA	L-L
							AC	
E5b.333	*elovl5b*	333/4	0.9071	0.4988	0.3744	0.4758	CC	N-N
							CT	
							TT	
E5b.424	*elovl5b*	424/4	0.4387	0.3587	0.2944	0.2342	CC	L-L
							CT	
							TT	
E5b.711	*elovl5b*	711/6	0.5576	0.435	0.3404	0.3197	CC	T-T
							TC	
							TT	
E5b.782	*elovl5b*	782/7	0.145	0.1345	0.1254	0.0725	GA	R-Q
							GG	
E5b.813	*elovl5b*	813/7	0.2193	0.2181	0.1943	0.1245	CC	N-N
							CT	
							TT	

* The base position of SNP related to the start codon in the reference mRNA.

**Table 2:** Association analysis of the PUFA contents in common carp.

Trait	Marker	Perm_*p* Value of GLM	MarkerR^2^	FDR of ANOVA	MM	Mm
C18:3n-3	E5b.172	4.44 × 10^−3^	6.90	1.29 × 10^−9^	0.84 ± 0.59 *	1.79 ± 1.46
C20:3n-3	E5b.172	2.01 × 10^−3^	3.96	5.61 × 10^−11^	0.24 ± 0.11	0.41 ± 0.2
	E5b.782	3.54 × 10^−2^	2.15	2.69 × 10^−4^	0.26 ± 0.13	0.38 ± 0.19
C20:4n-3	E5b.172	1.54 × 10^−3^	7.12	1.07 × 10^−11^	0.28 ± 0.11	0.46 ± 0.22
C20:5n-3	E5a.87	3.12 × 10^−3^	4.13	1.89 × 10^−7^	3.45 ± 1.46	7.57 ± 6.88
C22:5n-3	E5b.172	7.71 × 10^−3^	3.70	1.58 × 10^−8^	1.58 ± 0.82	2.61 ± 1.36
C18:2n-6	E5b.172	3.60 × 10^−4^	2.86	1.73 × 10^−15^	23.21 ± 5.75	37.02 ± 17
C20:3n-6	E5b.172	6.70 × 10^−4^	3.37	1.71 × 10^−13^	2.63 ± 1.12	4.82 ± 2.71
	E5b.782	3.51 × 10^−2^	4.48	2.50 × 10^−4^	2.83 ± 1.51	4.25 ± 2.53
C20:4n-6	E5b.172	3.67 × 10^−2^	2.10	4.06 × 10^−4^	8.11 ± 3.79	11.61 ± 8.04
C22:4n-6	E5b.172	7.23 × 10^−3^	3.44	1.21 × 10^−8^	0.65 ± 0.28	1.05 ± 0.62
	E5b.782	4.51 × 10^−2^	1.83	1.57 × 10^−3^	0.68 ± 0.33	0.97 ± 0.62
C22:5n-6	E5b.172	2.30 × 10^−3^	4.07	1.22 × 10^−10^	2.15 ± 1.2	4.23 ± 3.02
	E5b.782	3.46 × 10^−2^	1.83	2.26 × 10^−4^	2.32 ± 1.51	3.84 ± 3.06

Perm_*p* value of GLM: *p* value corrected with 100,000 permutations test. FDR of ANNOVA: the corrected *p* value of ANOVA using the FDR method. Marker R^2^: the explained percentage of PVs by markers. M: major allele; m: minor allele. * The content of each PUFA is displayed as the mean ± SD value.

**Table 3:** The explained percentage of PV for each PUFA by each genotype combination from four SNP loci in *fads2b* and *elovl5b* across 223 individuals.

PUFA	fads2b.751	fads2b.1197	E5b.172	E5b.782	Genotype Combination
C18:3n-3	5	NA	6.9	NA	33.6
C20:3n-3	13	4.3	3.96	2.15	32.73
C20:4n-3	11	5	7.12	NA	33.22
C22:5n-3	11	5	3.7	NA	22.63
C18:2n-6	NA	4	2.86	NA	54.95
C20:3n-6	11	4	3.37	4.48	37.59
C22:4n-6	7	NA	3.44	1.83	21.26
C22:5n-6	9	3	4.07	1.83	28.19
C18:4n-3	NA	NA	NA	NA	4.24
C18:3n-6	NA	NA	NA	NA	5.02
C20:5n-3	NA	NA	NA	NA	8.02
C20:4n-6	NA	NA	2.1	NA	13.06

The explained percentage of PV is represented as the Marker R^2^. NA means that this SNP is not associated with the PUFA content.

## Data Availability

The data presented in this study are available in supplementary material.

## Supplementary Materials

The following supporting information can be downloaded at: https://www.mdpi.com/article/10.3390/biology11030466/s1. Figure S1: Genetic relationship among three strains; Figure S2: Haplotype blocks in the coding regions of *elovl5a* and *elovl5b*; Table S1: The primers used for amplification of the coding sequence of *elovl5a* and *elovl5**b*; Table S2: *Elovl5a* genotypes; Table S3: *Elovl5**b* genotypes; Table S4: Genotype combination of two SNPs in *elovl5b* and two SNPs in *fads2b* and the corresponding PUFA contents for joint analysis; Table S5: Pearson correlation between the breeding values and the PUFAs contents based on all cSNPs in *fads2b* and *elovl5**b*.
