# Supplementary material for: Association Analysis between Genetic Variants of elovl5a and elovl5b and Poly-Unsaturated Fatty Acids in Common Carp (Cyprinus carpio)
Source: Biology (Basel). 2022 Mar 18;11(3):466. doi: 10.3390/biology11030466 (PMC8945013; doi:10.3390/biology11030466)

Figure S1. Genetic relationship among three strains.

(A). Principal component analysis of the FRL, HHL and JL common carp strains, which were colored with different plots. The first and second axes explain 41.61% and 10.76% of the variance, respectively. (B). Population genetic structures of three strains with five cluster numbers (K = 2~6). Each individual is showed with a vertical bar broken into different colored segments. Each color represents one ancestral population. The length of each colored segment shows the proportion of the ancestral population in one individual.

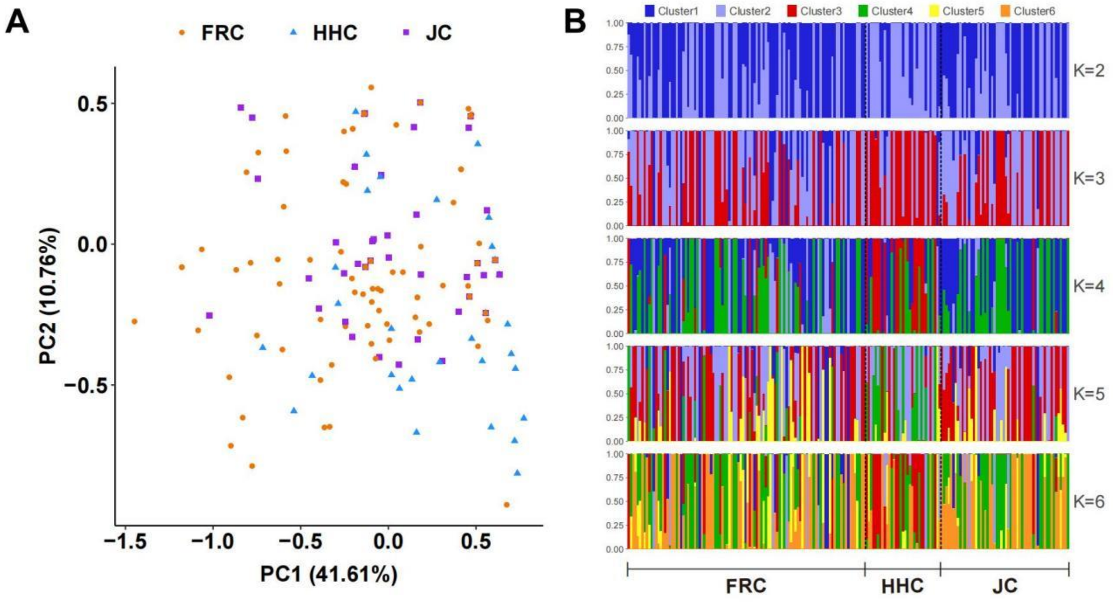

Figure S2. Haplotype blocks in the coding regions of *elovl5a* and *elovl5b*.

Linkage disequilibrium (LD) block structures in *elovl5a* (A) and *elovl5b* (B). The red color means tight linkage. The region surrounded with the black lines represents a haplotype block.

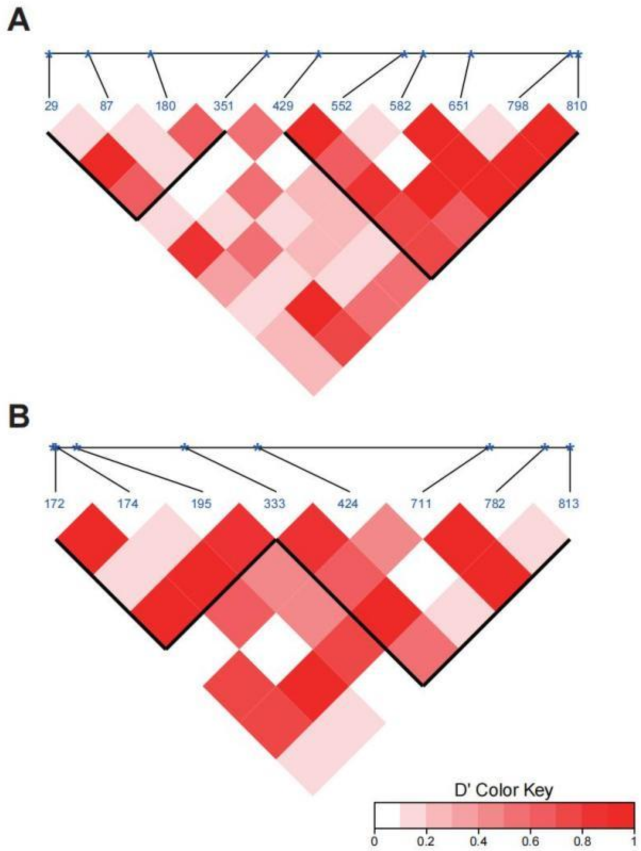

Supplement: Supplementary file 1 [file biology-11-00466-s001.zip › Supplementary-Figures.pdf]
